# Functional assays with human patient-derived enteroid monolayers to assess the human gut barrier

**DOI:** 10.1016/j.xpro.2021.100680

**Published:** 2021-07-17

**Authors:** Ibrahim M. Sayed, Courtney Tindle, Ayden G. Fonseca, Pradipta Ghosh, Soumita Das

**Affiliations:** 1 Department of Pathology, University of California, San Diego, CA 92093, USA; 2 Department of Cellular and Molecular Medicine, University of California, San Diego, CA 92093, USA; 3 HUMANOID CoRE, University of California, San Diego, CA 92093, USA; 4 Department of Medicine, University of California, San Diego, CA 92093, USA; 5 Moore’s Comprehensive Cancer Center, University of California, San Diego, CA 92093, USA; 6 Veterans Affairs Medical Center, VA San Diego Healthcare System, La Jolla, San Diego, CA 92093, USA

**Keywords:** Cell Biology, Cell culture, High Throughput Screening, Immunology, Microbiology, Stem Cells, Organoids

## Abstract

Here, we describe the use of polarized patient enteroid-derived monolayers (EDMs) to assess the impact of e-cigarettes on the human gut barrier. These EDMs can be adapted to culture in a 96-well plate for high-throughput screening. We model the effect of e-cigarettes by combining pathogens, enteroids, and e-cigarette vapor-infused media and assess gut barrier integrity, bacterial internalization, and inflammatory response of the gut epithelium. This protocol can be used to assess the effects of e-cigarette components on gut functions.

For complete details on the use and execution of this protocol, please refer to Sharma et al. (2021).

## Highlights

• Enteroid-derived monolayers (EDMs) are used for testing barrier integrity
• Polarized human EDMs are adapted for high-throughput screening
• E-cigarette vapor-infused media is added in the EDM model for testing
• EDMs are co-cultured with pathogens to study the innate immune response

## Graphical abstract

**Figure:**
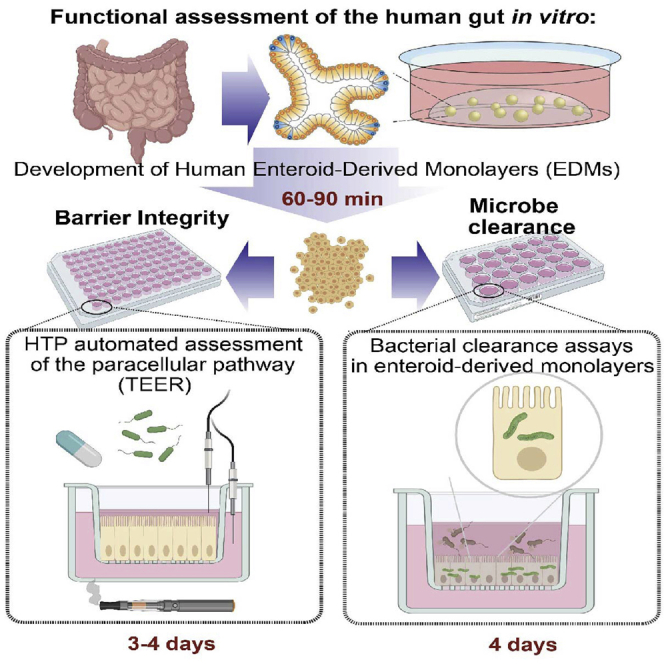

## Before you begin

Biopsies from healthy humans should be processed shortly after the sample collection. Biopsies should be preserved and transferred in wash media containing ROCK inhibitors on ice package. Crypt-derived stem cells were isolated from the colonic and ileal biopsies of healthy human subjects, grown as 3D organoids (Jung et al., 2011, Sato et al., 2011, Sato et al., 2009, Ghosh et al., 2020, Sayed et al., 2020a, Sayed et al., 2020c, Sharma et al., 2021).

**CRITICAL:** It is recommended to isolate the stem cells from the biopsies at the same day of collection. Isolation of the stem cells was also successful if it is done within 48 h of samples collection as long as the biopsies are preserved in the same condition of sample collection (i.e same transport media and ice package).

### Isolation of the stem cells from biopsies

**Timing: 60–90 min**

1. Digest colon or ileal tissues 2 mm in length using 1–2 mL of collagenase type I (2 mg/mL) containing gentamicin (50 μg/mL) dissolved in PBS at 37°C. Check the tissues every 10–15 min and do mechanical pipetting to speed the process of cell isolation. Stop the collagenase step when 50–80% of the tissues are digested and the stem cells are separated. This step requires about 30–40 min.
2. Inactivate the collagenase using at least an equal volume of wash media containing fetal bovine serum. The composition of washing media is mentioned in Material and Equipment section.
3. Filter the digested tissues using a 70-μm cell strainer to isolate single or small clusters of cells and to remove the tissue debris.
4. Wash the filtered cells using washing media, and then precipitate the washed filtered cell pellets by centrifugation at 200 xg for 5 min.
5. Embed the living stem cells into a basement membrane matrix (Matrigel) dome and cultured using 50% stem cell-enriched WRN conditioned media (WNT 3a, R-spondin, and Noggin) (prepared from L-WRN cells, ATCC CRL-3276^TM^) containing specific components such as Y27632 (ROCK inhibitor) and SB431542 (an inhibitor for TGF-β type I receptor), nicotinamide, A83-01 (an ACTIVIN /NODAL/TGF-β pathway inhibitor), Epidermal growth factor, and SB202190 (a p38 MAPK inhibitor) to support the growth and long-term cultures of 3D organoids (Jung et al., 2011, Sato et al., 2011, Sato et al., 2009, Lee et al., 2020). The concentration of the added inhibitors/ supplements is mentioned in Material and Equipment section. The media and supplements of human organoids were obtained from the HUMANOID CoRE (UC San Diego, CA, USA).

### Growing and expansion of 3D organoids

**Timing: 7–14 days**

6. Culture the stem cells in Matrigel domes on low binding tissue culture plates in a 37°C 5% CO2 incubator
7. For the first passages (p0-p2), use the splitting ratio range (1:1) to (1:3) in 24-well low binding tissue culture plates. Make the Matrigel dome by spreading 15 μL of Matrigel and single cell mixture per well and incubate the plate upside down for 5 min in a 37°C 5% CO2 incubator.
8. One day after the splitting, the organoids are small in size (Figure 1A), and the size and number of organoids increase with time by providing more fresh media containing the above mentioned supplements/inhibitors. When adequate amount of medium sized organoids (p0-p2) is developed in 24-well plate (Figure 1B), expand the organoids within the Matrigel in more wells in 24-well plate with the ratio (1:1 to 1:3) depending on the organoid number and size.

**Figure 1:**
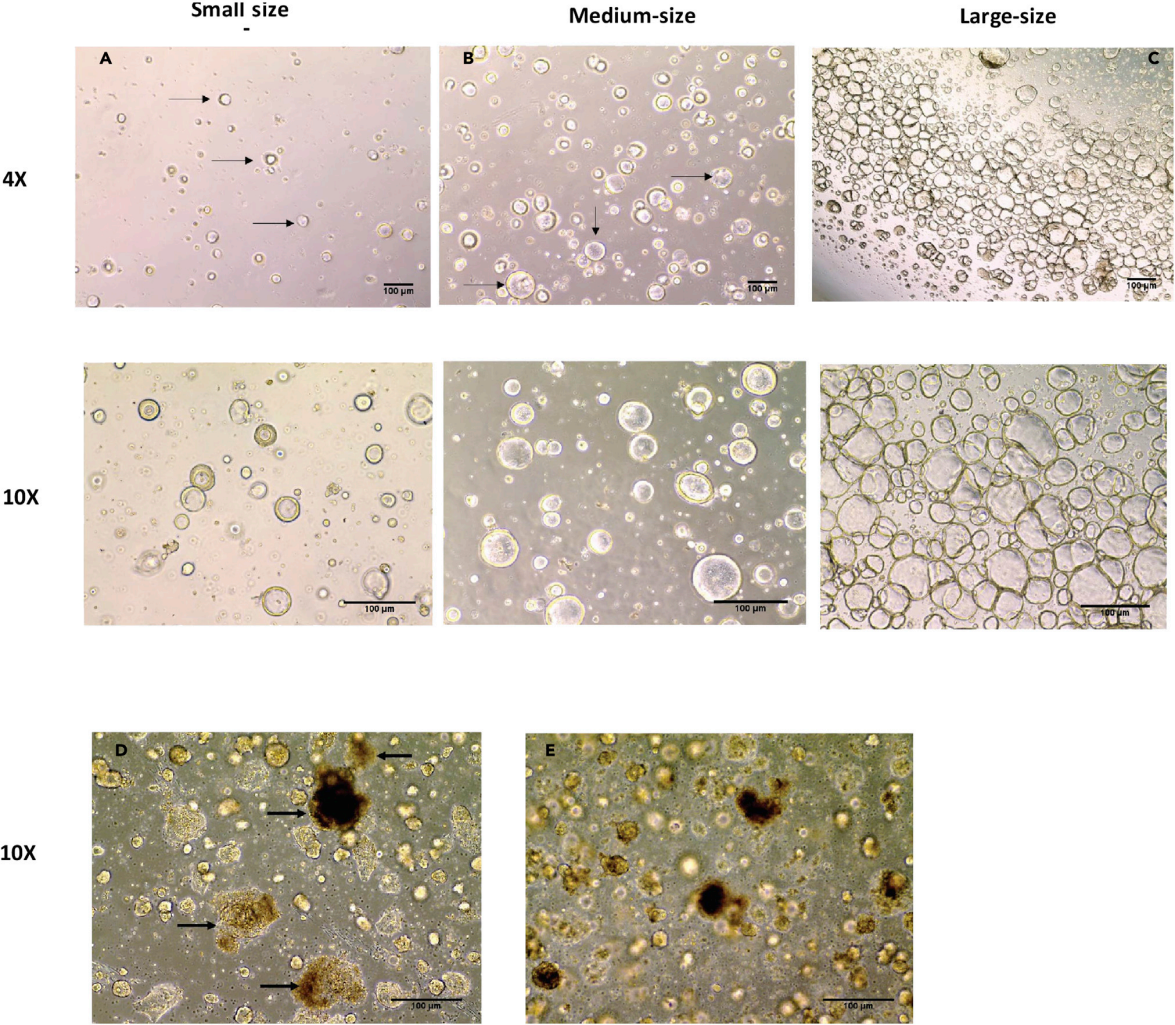
Representative images of different sized organoids, healthy and unhealthy organoids Day1 after the splitting**:** the organoids are small in size (A), the size and number of the organoids are increased with time till they become medium sized (B) and large sized (C). (A–C) represent examples of healthy organoids. Upper panel is 4× images and lower panel is 10× images (D and E) represent examples of unhealthy organoids, arrows point to the organoids where the black spots are accumulated as a sign of cell death. Representative Images are 10×. Scale bar 100 μm
9. Around passage 2 or 3 (p2-p3), expand the organoids from 24-well culture plate to 12-well culture plates. Split one to three wells of a 24-well to 1 to 3 wells of a 12 well low binding tissue culture plate depending on the organoid number and size. Spread 25μL of Matrigel and stem cell mixture per well and incubate upside down for 10 min in a 37°C 5% CO2 incubator (Jung et al., 2011, Sato et al., 2011, Sato et al., 2009).
10. After 6–7 days of growth, expand the organoids in 12-well low binding tissue culture plates using a (1:5) or (1:6) ratio when they become large sized (Figure 1C). Healthy and adequate amount of medium and large sized 3D organoids are required to prepare 2D EDMs.
11. Check the organoids daily for continued growth and that the cells are healthy (Figures 1A–1C) and not dying (dead organoids appear black) (Figures 1D and 1E). Change the organoid media with all supplements and inhibitors every 2–3 days.

**CRITICAL:** The growth of organoids, especially in the first few passages (after the isolation from the biopsies), requires special attention in determining the expansion (splitting) ratio. Using a high expansion ratio (for example 1:6 or 1:12) with poorly growing organoids in the first few passages (p0-p2) could lead to loss of the line.

## Key resources table

**Table:** 

REAGENT or RESOURCE	SOURCE	IDENTIFIER
**Bacterial and virus strains**		

Adherent Invasive *Escherichia coli* Strain LF82 (AIEC-LF82)	Arlette Darfeuille-Michaud, Inserm	Darfeuille-Michaud et al., 2004. (Darfeuille-Michaud et al., 2004)

**Biological samples**		

Human colon and ileal biopsies	HUMANOID CoRE, VA Hospital, San Diego	UCSD HRPP Project ID190105
L-WRN cells with Wnt3a, R-spondin, and Noggin.	The American Type Culture Collection (ATCC)	ATCC CRL-3276^TM^

**Chemicals, peptides, and recombinant proteins**		

Fetal bovine serum	Sigma-Aldrich	Cat# F2442-500ML
Dulbecco's Modified Eagle Medium/Nutrient Mixture F-12 containing 15 mM HEPES	Sigma-Aldrich	Cat#D6421-500ML
SB431542 (an inhibitor for TGF-β type I receptor)	Tocris a biotechne brand (R&D)	Cat# 1614/50
Y27632 (ROCK inhibitor)	Tocris (R&D)	Cat# 1254
Advanced DMEM/F12	Thermo Fisher Scientific	Cat# 12634028
TrypLE™ Select Enzyme (1×),no phenol red	Thermo Fisher Scientific	Cat# 12563011
Collagenase, Type I, powder	Thermo Fisher Scientific	Cat # 17101015
Corning® Matrigel® Basement Membrane Matrix, LDEV-free, 10 mL	Corning	Cat# 354234
Gentamicin (50 mg/mL)	Thermo Fisher Scientific	Cat# 15750060
EDTA (0.5 M), pH 8.0, RNase-Free	Thermo Fisher Scientific	Cat# AM9260G
Triton™ X-100	Sigma-Aldrich	CAS Number 9002 -93-1 X100-500ML.
Gibco™ Trypan Blue Solution, 0.4%	Thermo Fisher Scientific	Cat# 15-250-061
BD Difco™ LB Broth, Lennox	Thermo Fisher Scientific	Cat# DF0402-17-0
BD Difco™ Dehydrated Culture Media: LB Agar, Miller (Luria-Bertani)	Thermo Fisher Scientific	Cat# DF0445-17-4
Gibco™ DPBS, no calcium, no magnesium	Thermo Fisher Scientific	Cat# 14190250
SB 202190 (Inhibitor p38 MAP kinase)	Sigma-Aldrich	Cat# S7067
A-83-01 (TGF-beta inhibitor)	Tocris a biotechne brand (R&D)	Cat# 2939
Nicotinamide	Sigma-Aldrich	Cat# N0636
N-Acetyl-L-cysteine	Sigma-Aldrich	Cat# A9165
Gibco™ GlutaMAX™ Supplement	Thermo Fisher Scientific	Cat# 35050061
Animal-Free Recombinant Human EGF	PeproTech	Cat# AF-100-15
Nicotine	Sigma-Aldrich	Cat # 54-11-5
Propylene glycol	Sigma-Aldrich	P4347-500ML
Glycerin	Sigma-Aldrich	G2289-500ML

**Critical commercial assays**		

Quick-RNA Microprep Kit	Zymo Research	Cat# R1050

**Software and algorithms**		

Automated TEER Measurement System (REMS AutoSampler)	World Precision Instrument (WPI)	https://www.wpiinc.com/sys-rems-automated-teer-measurement-system

**Others**		

Corning HTS Transwell-96 Electrode	WPI	REMS-96C
Automated TEER Measurement System (REMS AutoSampler, version 6.02)	WPI	SYS-REMS
Replacement REMS HTS Electrode	WPI	Multiple SKUs
Countess II Automated Cell Counter	Thermo Fisher Scientific	AMQAX1000
Fisherbrand™ Sterile Cell Strainers	Thermo Fisher Scientific	Cat# 22-363-548
Transwell 96-well Permeable Support with 0.4 μm PET Membrane	Corning	Cat# 7369
Transwell 24-well Permeable Support with 0.4 μm PET Membrane	Corning	Cat# 3470
E-cigarette atomizer and the rechargeable battery	SCIREQ (Scientific Respiratory Equipment)	N/A
E-liquids including nicotine, propylene glycol, and vegetable glycerin	Xtreme Vaping	N/A

## Materials and equipment

### Preparation of human monolayer EDMs

**Table:** Preparation of primary culture media (100 mL) (Stored at 4°C for 1–2 months according to the expire date)

Reagent	Final concentration	Amount (mL)
Adv. DMEM-F12	NA	79
Fetal bovine serum (FBS)	20%	20
Glutamax 100× (200 mM)	2mM	1
Total		100

**Table:** Preparation of Human monolayer EDMs media (100 mL) (stored at 4°C for 2–4 days)

Reagent	Final concentration	Amount
Primary culture media	NA	90 mL
50% WRN conditioned media (CM) without any added cocktails	5%	10 mL
Y-27632 (ROCKi)(10 mM)	10μM	100 μL
N-acetyl cysteine (500 mM)	1mM	200 μL
Total		100 mL

***Note:***

The 50% CM media required for the preparation of human EDMs media should be without any inhibitors added.

The volume of human monolayer EDMs can be adjusted and the amount of ingredient added is modified accordingly.

The volume of human monolayer EDMs prepared should be determined according to the number of the transwells prepared. The EDM media should be fresh for each experiment.

### Preparation of 50 mL media with cocktails for the culture of organoids (50 mL)

**Table:** 

Components	Stock conc.	Volume	Final conc.
50% CM		49 mL	
Y-27632 (ROCKi)	10 mM	50 μL	10 μM
SB431542	10 mM	50 μL	10 μM
Glutamax	200 mM (100×)	250 μL	1 mM
N-acetyl cysteine (NAC)	500 mM	100 μL	1 mM
rh-EGF	100 μg/mL	25 μL	50 ng/mL
Nicotinamide	1M	500 μL	10 mM
A-83-01	2 mM	12.5 μL	500 nM
SB202190	30 mM	17 μL	10 μM

***Note:*** All the components are aliquoted and saved at –80°C till use. The volume of each aliquot/ component should be suitable for a single use to avoid freezing and thawing of the components.

**CRITICAL:** Repeated freezing and thawing of the aliquots should be avoided.

***Note:*** The preparation of all the added components (aliquots from each component are stored at –80°C for 2–3 months) mentioned in the table above is done as the following:

• NAC: to prepare 20 mL 500 mM: dissolve 1.64 g in 20 mL water in 50 mL tube, then filter the solution through 0.2 μM syringe filter and aliquot.
• Nicotinamide: to prepare 20 mL 1 M: dissolve 2.44 g in 20 mL water in 50 mL tube, then filter the solution through 0.2 μM syringe filter and aliquot.
• Y-27632 (ROCKi): prepare 10 mM solution by adding the appropriate volume of water according to the manufacturer's instruction at the website https://www.tocris.com/products/y-27632-dihydrochloride_1254?
• SB202190: to prepare 30 mM stock, add 2.51 mL of DMSO to 25 mg vial of SB202190 and make aliquots of 20 μL each.
• SB431542: prepare 10 mM solution by adding the appropriate volume of DMSO according to the manufacturer's instruction at the website https://www.tocris.com/products/sb-431542_1614?
• A83-01: prepare 2 mM stock by adding the appropriate volume of DMSO according to the manufacturer's instruction at the website https://www.tocris.com/products/a-83-01_2939?

### Washing media used for organoids and EDMs preparation (Stored at 4°C for few months according to the expire date)

**Table:** 

Reagent	Final concentration	Amount (mL)
Dulbecco's Modified Eagle Medium/Nutrient Mixture F-12	NA	440
Fetal bovine serum (FBS)	10%	50
Glutamax 100× (200 mM)	2mM	5
Penicillin/ Streptomycin 100×	1×	5
Total		500

**CRITICAL:** The washing media used in the infection experiments of EDMs does not include antibiotics.

### Preparation of e-cigarette vapor-infused media

The mixture of e-cigarette liquid contains 70% propylene glycol, 30% glycerol without additives and/or flavors. The preparation of e-cigarette vapor infused media is done with or without 6 mg/mL nicotine (Sharma et al., 2021, Alasmari et al., 2021, Alhaddad et al., 2020). E-cigarette vapor-infused media is generated by application of negative pressure on the activated battery, which causes the e-liquid to be heated and drawn through the internal atomizer and then a syringe including 10 mL of DMEM/F12 media containing 15 mM HEPES and 10% FBS. The infection media is developed by exposure to 50 mL of e-cig vapor generated from the vaporization of the e-cig liquid for 30 times, involving a 12-s interval between each exposure.

### Preparation of LB broth (Stored at 4°C for 1 month)

**Table:** 

Reagent	Final concentration	Amount
LB broth	20 g/L	20 g
Distilled Water	NA	1 L
Total		1 L

***Note:*** 20 g of LB broth includes 10 g of Tryptone, 5 g Yeast extract, and 5 g sodium chloride. Autoclave the LB broth solution at 121°C for 20 min, and allowed to cool down to room temperature around 20°C–22°C . Make aliquots of the broth in 50 mL tubes.

**Pause point:** The LB broth aliquots can be kept at 4°C for 2-weeks- 1 month.

### Preparation of LB agar plate

**Table:** 

Reagent	Final concentration	Amount
LB broth	NA	25 g
LB agar	1.5%	15 g
Distilled Water	NA	1L
Total		1L

***Note:*** Autoclave the LB agar solution at 121°C for 20 min, and then allowed to cool down until the solution is warm, but does not solidify. Bacterial culture tri-plates are prepared by pouring 6 mL of LB agar solution per tri-section. Leave the plates at room temperature around 20°C–22°C until the agar solidifies.

***Note:*** For the bacterial clearance assay, tri-plate is recommended over a single plate since it helps to test several bacterial dilutions from the same sample at the same time and same plate.

**Pause point:** The bacterial culture plates can be kept at 4°C for 2–4 weeks in the closed packet. At the time of use, the plate is kept at room temperature around 20°C–22°C for 10–15 min before adding the bacterial dilutions.

## Step-by-step method details

### Part 1: Development of human EDMs in 96-well inserts

**Timing: 3–4 days**

Day 0 of the EDMs preparation

1. Prepare the Human monolayer EDM media required for the differentiation of the stem cells to colon specific cells. The EDM model resembles the physiologic gut lining in which all cell types (enterocytes, goblet, Paneth enteroendocrine, and tuft cells) are proportionately represented (Sato et al., 2011, Sato et al., 2009, Sayed et al., 2020a, Miyoshi and Stappenbeck, 2013).
  a. The components of the human monolayer EDM media is mentioned in the previous section under the title “Materials and Equipment.”
  b. Allow media to incubate at room temperature around 20°C–22°C during the step of EDMs preparation. Since the step of EDM preparation takes 30–45 min, the EDM media can be kept at room temperature around 20°C–22°C at the start of EDM preparation.

**CRITICAL:** The human monolayer media should be fresh, constituted on the day of EDMs preparation. The media can be kept at 4°C for few days (2–3 days). Storing the media at 4°C for a longer period could affect the degree of stem cell differentiation due to the stability and efficacy of the components added to human monolayer EDM media could be affected by long-term storage.

2. Prepare Matrigel solution diluted in phosphate-buffered saline (PBS) at a (1:40) ratio (around 300 μg/mL) on ice.
  a. Coat each transwell with 50 μL of the PBS-diluted Matrigel.
  b. Leave the plate at room temperature around 20°C–22°C in the hood during the period of monolayer preparation (60–90 min).

**CRITICAL:** Dilute the Matrigel in PBS without EDTA. Avoid introducing bubbles in the Matrigel PBS mixture.

3. Splitting of 3D organoids into single cells
  a. Remove media from the organoid culture grown in 12-well plate, then add 1 mL of 0.5 μM PBS-EDTA to each well.
  b. Scrape cells/Matrigel in PBS-EDTA mixture and collect the cell suspension in a 15 mL conical tube, then centrifuge at 200 xg for 4 min to precipitate the cell pellets. No wash media is used in this step.
  c. Remove the supernatant above the pellets and add 300 μL of TrypLE / well to separate the cells from each other. The volume of TrypLE should be adjusted according to the numbers of wells collected
    i. Incubate the TrypLE suspension in a 37°C water bath for 5–15 min.
    ii. Disrupt the organoids mechanically in the TrypLE mixture by pipetting the cells up and down up to 20 times using a p1000 micropipette to have single or small clusters of cells. Pipetting should be continued until no cell clumps/ aggreagtes are appeared.
    iii. Inactivate the TrypLE by adding a serum-containing buffer such as wash buffer at the ratio (1:5) (1 TrypLE: 5 washing media).
    iv. Centrifuge at 200 × *g* for 4 min to remove the TrypLE and precipitate the dissociated cells.

**CRITICAL:** The time of TrypLE incubation depends on the density, size, and overall health of the organoids. Large and dense organoids require a longer incubation period with the TrypLE at 37°C. Inadequate incubation of organoids with the TrypLE or insufficient pipetting of the organoids could lead to incomplete dissociation of organoids into single stem cells. Alternatively, clumps of condensed cells are formed, which could affect the differentiation of the stem cells after that.

4. Filtration and counting the single cells,
  a. Place a 70 μm filter over a 50 mL tube. Wet the filter with 200 μL human monolayer media (prepared at step 1).
  b. Resuspend the cells in the desired volume of human monolayer media to get a good concentration of cells and to allow complete resuspension of the cell pellet to avoid loss of cells in the filter (∼200 μL per transwell).
  c. Filter the suspended cells using the pre-wet 70 μm filter over a 50 mL tube.
  d. Count the single cells or very small clusters of cells using the Trypan blue method or automated counter.

**CRITICAL:** The human monolayer media should be kept at room temperature around 20°C–22°C before the suspension of single cells.

**CRITICAL:** The volume of human monolayer media required to dissolve the single cells should be appropriate, 1 mL is a good start. If the concentration of cells is high, more media can be added till it reach the required concentration of cells.

**CRITICAL:** If the cells are more diluted in human monolayer media, the cells can be precipitated again by centrifugation at 200 × g and then dissolved in the appropriate volume.

5. Seeding the single cells in the transwell (Figure 2; Methods video S1)
  a. Calculate the number of cells needed for seeding 8 ×10^4^ cells per transwell. Prepare the appropriate mixture of cell suspension and monolayer media needed.
  b. Carefully aspirate the PBS from Matrigel coating in all wells without touching the tip to the bottom of the well.

**Figure 2:**
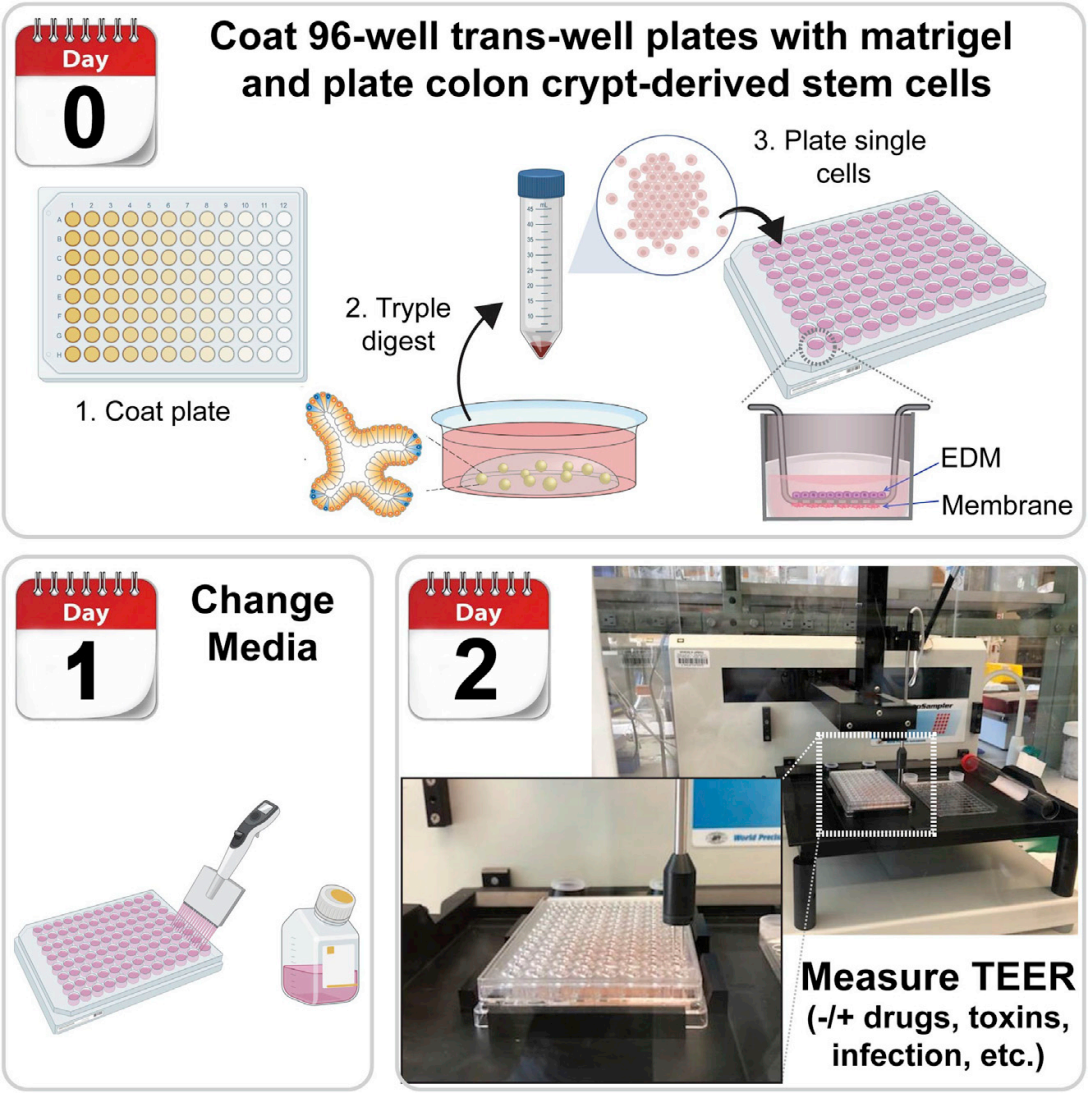
Development of human EDMs in 96-well inserts Day 0: Coat 96-well trans-well plates with Matrigel and plate stem cells Day1: Media change Day2: Measure the TEER by REMS AutoSampler and start the experiment.
6. Add 250 μL of monolayer media to the basolateral side of the transwell. Add 100 μL of the cell suspension mix to the apical side of each well. Incubate the plate on a flat surface inside the hood for ∼15 min. Then carefully transfer to the incubator on a flat shelf.

Methods video S1. The Development of high-throughput functional assay platform with human patient-derived enteroid monolayer and measurement of the TEER by REMS AutoSampler, related to part 1: development of human EDMs in 96-well Inserts, steps 1–9 Also the video includes part 2-PC-controlled high-throughput automated TEER measurement for epithelial monolayer in 96-well plate (REMS AutoSampler), steps 18–24.

Day 1 of the EDMs preparation (Figure 2)

7. Change the media from the apical and basolateral sides of the transwells using the monolayer media.
8. Check the monolayer appearance under the microscope. After 24 h, you should see the cells start to flatten out, but all space between cells may not be filled until 48 h post-seeding.
9. Measure the transepithelial electrical resistance (TEER) using the REMS automated TEER machine (as will be discussed in detail in the next step). The TEER value after 24 h (first-time point) helps to follow the TEER kinetics of the monolayers at different time points, which indicates the monolayer's efficiency (Figure 2).

**CRITICAL:** The human monolayer media should be kept at around 20°C–22°C before changing the media and the media should be added smoothly and slowly on the apical side of the transwells (inserts) to avoid the detachment of the differentiated cells from the transwells.

Day 2 of the EDMs preparation (first exposure to vapor infused media)

10. Measure the TEER in the morning using the REMS automated TEER machine before applying any conditions.
11. Expose the basolateral side of the transwells to the e-cigarette vapor-infused media for 4 h. Single exposure of the EDMs with the vapor media for 4 h represents acute exposure)(Sharma et al., 2021). To mimic the vapor exposure to the gut, we expose the basolateral side of EDMs (which represents the gut epithelium side facing the blood), while the apical side of the EDMs represents the side of epithelium facing the internal GIT.
12. Collect supernatants from the basolateral side of the transwells at 4 h of exposure to assess the level of inflammatory cytokines.
13. Collect the supernatant from the apical part of the transwells, save the supernatant at –20°C until the analysis. The supernatant collected from apical and/or basolateral compartments can be used to assess the cytokine levels and other functional assays.
14. Collect the RNA lysate to assess the transcriptome change upon addition of e-cigarette vapor-infused media, Add RNA lysis buffer to the cells attached to the transwells, incubate the lysis buffer with the transwell for 2–3 min, then pipette the lysis buffer up and down 10 times and then collect the cell lysate in 1,5 Eppendorf tubes for further RNA extraction.

**Pause point:** The cell lysate in RNA lysis buffer is kept at –80°C till the step of RNA extraction. Supernatant collected can be kept at –20°C or –80°C till analysis for cytokine assay.

Day 3 of the EDMs preparation (chronic exposure for vapor infused media)

15. To assess the effect of chronic exposure of EDMs to e-cigarette vapor-infused media, expose the EDMs on Day 3 of the preparation continuously to e-cigarette vapor-infused media for 4 h, and the medium is refreshed with new e-cigarette vapor-infused media for repeated exposure (3 times exposure) prior to analyzing them at 24 h (Day 4 of EDMs preparation). Measure the TEER after 24 h for exposure.
16. Collect supernatants from the basolateral side of the transwells at 24 h of exposure to the vapor-infused media.to assess the level of inflammatory cytokines.
17. Collect the cell lysate for RNA extraction as described in the previous step.

**Pause point:** The cell lysate in RNA lysis buffer is kept at –80°C till the step of RNA extraction. Supernatant collected can be kept at –20°C or –80°C till analysis.

**CRITICAL:** In 96-well plate, the RNA extracted from one well is relatively low and insufficient to assess the transcriptome analysis. Therefore, 2–3 wells from each condition should be combined to get an adequate RNA concentration for qPCR and/or sequencing analysis.

**CRITICAL:** After RNA extraction and measurement of the RNA concentration, aliquot the fresh RNA into 2 parts: 200–300 ng for RNA sequencing and 500 ng–1000 ng for transcriptome analysis by qRT-PCR. Repeated freezing and thawing for the extracted RNA should be avoided.

***Note:*** The EDMs used in chronic exposure experiment (Day 3) are not the same ones used for acute exposure experiment (Day 2), but they are derived from the same organoid cultures.

### Part 2: PC-controlled high-throughput automated TEER measurement for epithelial monolayer in 96-well plates (REMS AutoSampler)

**Timing: 5–60 min, depending on the numbers of wells to be measured**

18. Place the plate on the REMS plate holder and ensure all 4 corners are properly placed and clipped in (Figure 2 and Methods video S1).
19. Select the option “Take Data/ Well Sequence” tab in REMS software: Perform 70% Ethanol Rinse (Wash Station 1: Right) followed by Wash Media Rinse (Wash Station 2: Left).
20. Select “Stabilize” to confirm that the Recording Electrode is working correctly. Range should be within 32–45Ω and varies with different media composition.
21. Using the REMS Autosampler software, select the Plate Files Tab -> Edit Plate File. Using the Plate File Settings window, Perform calibration test of A1 Well using the WPI REMS-96C recording Electrode.
  a. Under the Movement tab: Send Electrode to well A1 Position using the XY Position Axis.
  b. Adjust Z Low to 130 mm and ensure basal Electrode is centered (Adjust XY as needed).
  c. Send Z Low down 2 mm at a time to confirm basal electrode calibration (no edge-clipping and relatively centered).
  d. Close the Plate File Settings without saving the adjustments to prevent overwriting the original settings
22. Return to “Take Data/ Well Sequence” tab. Set the TEER Electrode Sequence:
  a. Check the increment box and fill in the number 1 to start your sequence at 1.
  b. Go to the plate map to select the targeted wells. Set the desired sequence for TEER measurements. The Electrode is still centered overA1 well during these steps.
23. If there are multiple conditions, select “Data Collection” tab and select Rinse Between Wells.
24. Label the experiment name and select “Start” under “Take Data/ Well Sequence” tab. TEER values will be saved in a Text File.

**CRITICAL:** Calibration steps are important to ensure the electrodes will be correctly centered. Inappropriate calibration could lead to incorrect direction and movement of the Electrode, which could lead to the damage of expensive electrode.

**CRITICAL:** The electrode returns home (the original position) after every run, and the A1 calibration steps should be run every time the electrode returns to home. The abrupt mechanical movement made when the probe returns home can cause a slight misalignment of the XY position.

**Pause point:** It is recommended to coat the unused wells with plastic adhesive sheets for future use without causing contamination of these wells in the current analysis.

### Part 3: Gentamicin protection assay to assess bacterial internalization in the human EDMs

**Timing: 4 days**

**Figure 3:**
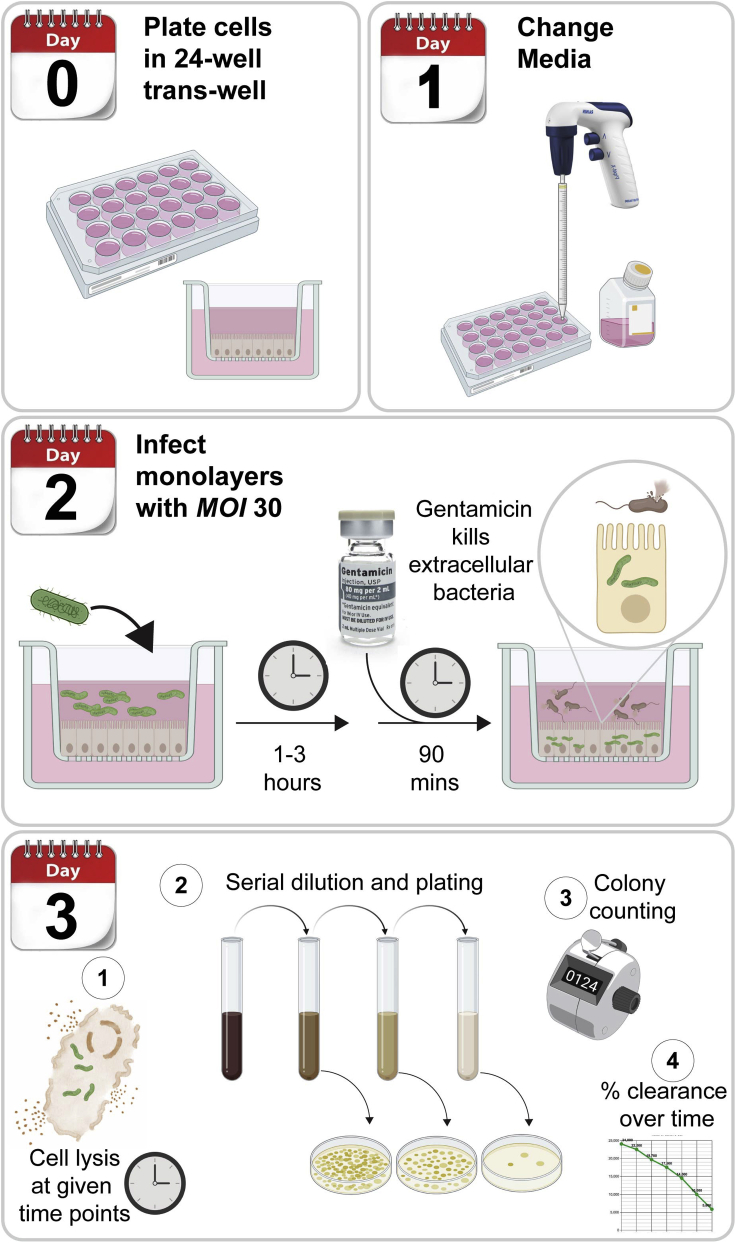
Gentamicin protection assay to assess bacterial internalization in the human EDMs Day 0: Plate cells in 24-well trans-well Day1: Media change Day2: Infect the EDMs with *E.coli* for 1–3 h then add gentamicin to kill extracellular bacteria. Lyse the cells and prepare the bacteria dilutions and plate Day3: Count the bacteria and replate the dilutions if needed

Day 0: Prepare the human monolayer EDMs as in protocol #1 in 24-well transwell (inserts) (Figure 3).

25. Follow the same steps in the human EDMs preparation as Part 1, starting from step 1 to step 4.
26. Plate 2 × 10^5^ cells per well in 24-well corning transwell inserts.
  a. The transwell inserts are coated with 130 uL of PBS-diluted Matrigel (1:40 dilution) with a dilution of 300 μg/mL.
  b. Add 700 μL of monolayer media to the basolateral side of the transwell. Add 200 μL of the cell suspension mix to the apical side of each well.
  c. The precautions and critical steps applied to the preparation of human EDMs in 96-well plate should also be applied in the preparation of EDMs in 24-well plate.

Day 1:

27. Change monolayer media on the apical and basolateral sides as described in protocol # 1
28. Culture *E. coli* LF82 in LB broth (Sayed et al., 2020a). We did the following steps :
  a. Make a day culture: inoculate one loopful of bacteria from an agar plate into 10 mL of LB in a 50 mL conical tube with a slightly loose lid to allow aeration. Incubate the culture at 37°C in an orbital shaker at 150 rpm for 6–8 h.
  b. Make overnight culture: Add 100 μL of the day culture to 10 mL of LB in a 15 mL conical tube. Incubate at 37°C without shaking for overnight (about 16 h) and the lid should be tightly closed to allow minimum aeration and keep the bacteria invasive.

Day 2: Infection of EDM exposed to e-cigarettes with *E.coli* LF-82

29. Expose the EDMs to the e-cigarette vapor-infused media and *E.coli* inoculum.

Expose the basolateral side of the transwells to the e-cigarette vapor-infused media for 4 h (single exposure) or 3 times, each 4 h apart prior to analyzing them at 24 h (chronic exposure).

30. Measure the OD_600_ of the *E.coli* LF-82 bacteria using a spectrophotometer.
31. Spin down the calculated concentration of bacteria for 2 min at 9391 xg. Aspirate the supernatant and resuspended the bacteria in 1 mL of wash media without antibiotics.
32. Infect the monolayers with ***E.coli* LF-82** at a multiplicity of infection (MOI) of 1:30 in wash buffer without antibiotics. Add 200 μL of the bacterial mixture to the apical side of each transwell.

**CRITICAL:** The infection media should be without antibiotics.

33. Kill the extracellular bacteria and lyse the cells for bacterial counting
  a. After 1–3 h of infection, add 200 μg/mL of gentamicin to kill the extracellular bacteria.
  b. After 90 min of incubation with gentamicin, remove the media from the wells used for the bacterial internalization at every time point. Add 150 μL of 1% Triton X-100 to the apical side of each transwell and incubate for 10 min in a 37° C 5% CO2 incubator.
  c. Lyse the cells by pipetting up and down 5–7 times. Do serial dilution of the cell lysate by adding 100 μL of lysate to 900 μL of 1× PBS (10^−1^ dilution). Dilutions up to 10^−6^ should be done depending on the cells.
    **CRITICAL:** Pipette the cell-lysis buffer cautiously to avoid the puncture of the membrane of the transwell.
  d. Plate 50 μL of 10^−6^, 10^−5^, 10^−4^, 10^−3^, 10^−2^, 10^−1^ dilutions on LB agar tri-plates. Place the tri-plates upside-down in a 37°C incubator for 16–18 h.
    **CRITICAL:** For longer infection time-points, remove the 200 μg/mL gentamicin after 90 min and add 200 μL of low gentamicin (50μg/mL) to the apical side of the wells till the end of the experiment.

Day 3: Count the colony-forming units (CFU) of bacteria

34. Count the colonies from different dilutions on the next day and calculate the average total CFUs.

## Expected outcomes

**Evaluation of the efficiency of the prepared EDMs in the 96-well plate:** Following the steps of EDMs formation, Sharma et al. showed that the stem cells are differentiated into polarized enteroid derived monolayer with apical and basolateral sides that physiologically mimic the in vivo gut lining (Sharma et al., 2021). The EDMs include all intestine cell types (enterocytes, goblet, Paneth enteroendocrine, and tuft cells) that are proportionately represented as shown by other groups and us (Sato et al., 2011, Sato et al., 2009, Miyoshi and Stappenbeck, 2013, Noel et al., 2017, Ghosh et al., 2020, Sayed et al., 2020a, Sayed et al., 2020c, Sayed et al., 2020b). Several readouts indicate the differentiation of stem cells into polarized epithelial cells **(*a) TEER kinetic by REMS*:** the TEER value at day 1 (day of EDM preparation) is low (undifferentiated stem cells), after that the TEER value is increased gradually in day 2 and day 3 indicating the formation of tight junctions among the differentiated epithelial cells. (***b) Assessment of the differentiation and stemness transcripts by qRT-PCR:*** The transcript level of stemness marker (Lgr-5), and differentiation transcripts (carbonic anhydrase and sucrase isomaltase for enterocyte markers, chromogranin for enteroendocrine marker, β-defensin and lysozyme for Paneth cell marker, DCLK-1 for tuft cell marker, and Muc-2 for goblet cell marker) is measured in the differentiated EDMs and compared to undifferentiated stem cells. Compared to undifferentiated stem cells, the differentiated EDMs show a significant reduction in the stemness marker and a marked increase in the differentiation markers after 48–72 h of EDM preparation. (**c) Immunofluorescence labeling of differentiation markers in the polarized EDMs.** As the the EDMs are well differentiated, the expression of stemness markers (Lgr-5) is reduced in differentiated EDMs compared to undifferentiated EDMs. While the expression of differentiation markers (Villin, Carbonic anhydrase, MUC2) is increased in the differentiated EDMs. Moreover, the expression of tight junction proteins such as occludin and E-cadherin is increased in the differentiated EDMs

**Test the impact of e-cigarettes on the integrity of the gut barrier**: E-cigarette disrupts the integrity of the human gut barrier and triggers inflammation (Sharma et al., 2021). We evaluated the impact of e-cigarette vapor exposure of the human EDMs after a single and repeated exposure. An acute single exposure to e-cigarettes was associated with a significant drop in TEER at 4 h, but the initial drop at 4 h was reversed after 24 h. Exposure of the EDMs to repetitive e-cigarettes exposures (3 times, resembles chronic exposure) caused a sustained drop in TEER at 24 h compared to control cells. There was a 3-fold increase in the percentage of burst tight junctions (TJs) TJs in the chronically exposed EDMs compared to control cells. Also, there was a significant drop in gene expression of ZO1 after chronic repetitive multiple exposures.

### Effect of e-cigarettes on the bacterial internalization and inflammatory response of the gut epithelium

Next, we assessed the impact of e-cigarettes exposure on gut reactivity to pathogens. Since e-cigarette disrupts the integrity of the human gut and increases leakiness (Sharma et al., 2021), we expected that the number of internalized bacteria is increased after repeated exposure to e-cigarette vapor. A single exposure to e-cigarettes vapor resulted in a slight increase in the number of internalized bacteria compared to control cells. While EDMs repeatedly exposed to e-cigarettes (3×) showed a statistically significantly higher number of internalized bacteria compared with control EDMs after 3 h of infection, suggesting higher infectivity of gut epithelium after e-cigarette exposure. Also, the number of internalized bacteria was increased after repetitive exposure to e-cigarettes compared to a single exposure to e-cigarette vapor.

## Limitations

Although the EDMs models mimic the *in vivo* physiological gut epithelium, one limitation of this model that it cannot be passaged and propagated. Since the 2D EDMs are derived from 3D organoids, there could be a batch-to-batch variation in the differentiated EDMs. Some components in the 3D organoid media such as Matrigel, fetal calf serum, growth factors, and inhibitors (mentioned in the table for preparation of 50% CM media) affect the proliferation of stem cells in the 3D organoids and hence could affect the differentiation of these cells into 2D EDMs especially after long maintenance of the stem cells on culture (Ji and Wu, 2020, Drost and Clevers, 2018). Therefore, the generation of 2D EDMs requires highly trained skills and regular quality controls. Furthermore, the abundance of differentiated cell types could slightly vary across independent biological replicates. Moreover, the development of 3D organoid and 2D EDMs are costly and require highly funding sources that are not matched with many research laboratories worldwide. Also, the EDM model mimics the gut epithelium, but coculture of the EDMs with multiple components such as immune cells, microbes, and vascular endothelial cells at the same time to mimic the gut environment is still challenging.

## Troubleshooting

### Problem 1

Poorly Differentiated EDMs (steps 8–10)

The prepared EDMs are poorly differentiated; this can be verified in the following conditions: (a) The TEER value is very low and has not changed after the EDMs preparation till 72 h post preparation. (b) The stemness marker and differentiation markers are comparable in the undifferentiated cells and EDMs. Although the measurement of TEER in 96-well plate is automatic, the preparation of EDMs is done manually.

Several causes could lead to poorly differentiated EDMS: (1) Unhealthy organoids from which the EDMs are generated; (2)An insufficient number of cells embedded in the transwell; (3) Old human monolayer EDMs media; (4) The wrong formulation of human monolayer EDMs media.

### Potential solution

Solution 1: Generate EMDs from healthy organoids. Organoids with numerous black centers and disintegrated organoids are not good sources for differentiated EDMs. To reduce the development of unhealthy organoids: a) Regularly change the organoid media, especially when the media color change to yellow which is a marker for the accumulation of toxic metabolite and therefore the media should be changed. b) Passage/ Split the organoids when they reach confluency. Use the correct splitting ratio. Growing of the organoid should be done in 50% CM that favors the stemness. c) Do not use 50% CM media stored for at 4°C for more than a week. d) Be sure that 50% CM media include all the cocktail components as mentioned in the Materials and Equipment section.

Solution 2: Add an adequate number of cells in the transwell. In 96 well plate: add 6 ×10^4^ to 1×10^5^ cell/ transwell. In 24 well plate: add 2×10^5^- 4×10^5^ cell/ transwell

Solution 3: Prepare the human monolayer EDMs media fresh.

Solution 4: Constitute the human monolayer EMDs correctly. Most of the components added to the 3D organoids are not required to the 2D EDMs, and their presence could affect the development of differentiated polarized EDMs.

### Problem 2

*E.coli LF82* did not infect the EDMs well (steps 29–32)

The prepared EDMs are differentiated well, but the infection of EDMs with the bacteria did not lead to the expected findings. This problem can be raised due to the following reasons: (1)Incorrect moi used; (2) Use old bacteria culture; (3) Decrease the bacterial infectivity due to the temperature, and the OD.

### Potential solution

Solution 1: Use the correct moi (1:30) to infect the EDMs, as the number of bacteria added depends on the number of cells added to the transwell.

Solution 2: The bacteria should be fresh, old bacteria do not replicate and be invasive enough in the EDM model. Also, the bacteria inoculum present in LB agar (source of infection) should not be older than 1 month. Confirm the bacteria growth on LB broth by measuring the OD600 of bacteria.

Solution 3: All infective steps should be performed with prewarmed material at 37°C. Optimal OD should be reached before infection (lower or higher OD influence the bacterial behavior and can affect the infectivity).

### Problem 3

Variation in the colony-forming units (CFU) produced from different dilutions of the same samples (steps 33–34)

The serial dilutions of the cell lysate containing bacteria (10^−1^ to 10^−6^) are done to assess the CFU. Some dilutions could give an inaccurate estimate of the actual bacterial cfu. This problem is probably due to errors in the preparation of bacterial serial dilutions.

### Potential solution

Solution 1: Experiment should be performed in duplicate and repeated in case of incoherent results.

Solution 2: Serial dilutions should be performed with well calibrated pipettes, the solution should be homogenized and tips should be changed between each dilution steps.

## Resource availability

### Lead contact

Further information and requests for resources and reagents should be directed to and will be fulfilled by the lead contact, Soumita Das (sodas@health.ucsd.edu)

### Materials availability

EDMs generated in this study will be made available on request, but we may require payment and/or a completed Materials Transfer Agreement if there is potential for commercial application.

### Data and code availability

This study did not generate/analyze a new dataset.
